# H4K20me3 co-localizes with activating histone modifications at transcriptionally dynamic regions in embryonic stem cells

**DOI:** 10.1186/s12864-018-4886-4

**Published:** 2018-07-03

**Authors:** Jian Xu, Benjamin L. Kidder

**Affiliations:** 1 Department of Neurology, Wayne State University School of Medicine, Detroit, MI USA; 2 Department of Oncology, Wayne State University School of Medicine, Detroit, MI USA; 3 Karmanos Cancer Institute, Wayne State University School of Medicine, Detroit, MI USA

**Keywords:** Embryonic stem cells, Bivalent, H4K20me3, H3K4me3, Epigenetics, Chromatin, Sequential ChIP-Seq, Pausing, RNA polymerase

## Abstract

**Background:**

Bivalent chromatin domains consisting of the activating histone 3 lysine 4 trimethylation (H3K4me3) and repressive histone 3 lysine 27 trimethylation (H3K27me3) histone modifications are enriched at developmental genes that are repressed in embryonic stem cells but active during differentiation. However, it is unknown whether another repressive histone modification, histone 4 lysine 20 trimethylation (H4K20me3), co-localizes with activating histone marks in ES cells.

**Results:**

Here, we describe the previously uncharacterized coupling of the repressive H4K20me3 heterochromatin mark with the activating histone modifications H3K4me3 and histone 3 lysine 36 trimethylation (H3K36me3), and transcriptional machinery (RNA polymerase II; RNAPII), in ES cells. These newly described bivalent domains consisting of H3K4me3/H4K20me3 are predominantly located in intergenic regions and near transcriptional start sites of active genes, while H3K36me3/H4K20me3 are located in intergenic regions and within gene body regions of active genes. Global sequential ChIP, also termed reChIP-Seq, confirmed the simultaneous presence of H3K4me3 and H4K20me3 at the same genomic regions in ES cells. Genes containing H3K4me3/H4K20me3 exhibit decreased RNAPII pausing and are poised for deactivation of RNAPII binding during differentiation relative to H3K4me3 marked genes. An evaluation of transcription factor (TF) binding motif enrichment revealed that DNA sequence may play a role in shaping the landscape of these novel bivalent domains. Moreover, H3K4me3/H4K20me3 and H3K36me3/H4K20me3 bound regions are enriched with repetitive LINE and LTR elements.

**Conclusions:**

Overall, these findings highlight a previously undescribed subnetwork of ES cell transcriptional circuitry that utilizes dual marking of the repressive H4K20me3 mark with activating histone modifications.

## Background

Embryonic stem (ES) cells exhibit the ability to self-renew indefinitely in culture and to differentiate into all cell types. While epigenetic regulation of chromatin plays a central role in controlling gene expression programs in ES cells, how ES cells maintain pluripotency is still a core question in stem cell biology. Posttranslational modification of histones, including methylation of histone 3, lysine 4 (H3K4), is thought to contribute to the regulation of ES cell self-renewal and pluripotency by regulating chromatin structure [1], marking active gene regulatory networks, and influencing the transcriptional state of the underlying DNA sequencing. Pluripotency regulators and genes highly expressed in ES cells are enriched with H3K4 methylation at transcriptional start sites (TSS) [2].

Previous work has also suggested that the repressive histone 3, lysine 27 trimethylation (H3K27me3) mark co-localizes with the activating H3K4me3 mark at developmental genes in ES cells [3]. Genes with H3K4me3/H3K27me3 bivalent domains are thought to be poised for activation upon differentiation, where H3K27me3 marks silence developmental genes in ES cells, and H3K4me3 marks poise genes for transcriptional activation during differentiation. However, an evaluation of H3K4me3 levels during ES cell differentiation suggests that H3K4 methylation is demethylated at H3K4me3/H3K27me3 bivalently marked genes during early differentiation [4–6] and re-established later in differentiation [5]. Our previous results demonstrate that H3K4me3 levels at promoters decrease on a global level following 3 days of ES cell differentiation [4]. In addition, analysis of H3K4me3 ChIP-Seq from two additional studies also revealed decreased H3K4me3 at H3K4me3/H3K27me3 bivalently marked genes during the initial stages of ESC differentiation [5, 6]. Therefore, because H3K4me3 is not maintained at bivalently marked chromatin during the initial stages of differentiation, and are only re-established at developmental genes during lineage-specific differentiation, the role for the H3K4me3/H3K27me3 bivalent domain in ES cells remains largely unknown.

Bivalent domains have also been identified in adult stem cells (mesenchymal stem cells) and lineage-committed preadipocytes, where H3K4me3 was found to co-localize with the repressive H3K9me3 histone modification at adipogenic master regulators [7]. While these results suggest that the histone 3, lysine 9 trimethylation (H3K9me3) heterochromatin mark pairs with the activating H3K4me3 mark in adult stem cells, it is unknown whether histone 4, lysine 20 trimethylation (H4K20me3), which is also enriched at heterochromatin regions, co-localizes with H3K4me3 in ES cells. H4K20 methylation is associated with several cellular processes including heterochromatin formation, transcriptional regulation [8], DNA damage repair [9, 10], DNA replication [11], chromosome condensation [12], and genome stability [10, 13]. While H4K20me1 is found in active genes [2, 14], H4K20me3 is thought to be a repressive histone modification, where H4K20me3 is associated with the formation of pericentric hetereochromatin, and H4K20me3 marks have been shown to repress transcription of repetitive elements [10, 15, 16].

Here, we show that H4K20me3 pairs with activating histone modifications H3K4me3 and RNA polymerase II (RNAPII) at transcriptional start sites (TSS) and co-localizes with H3K36me3 in gene body regions of actively transcribed genes in ES cells. Strikingly, while conventional H3K4me3/H3K27me3 bivalent domains mark developmental genes that are repressed in ES cells but poised for activation upon differentiation, the novel H3K4me3/H4K20me3 and H3K36me3/H4K20me3 bivalent domains described in this study mark active genes in ES cells. Moreover, H4K20me3/H3K4me3 marked genes display decreased RNAPII pausing and are poised for deactivation of RNAPII binding during differentiation. This newly described bivalent domain constitutes a subnetwork of the ES cell transcriptional circuit and provides insight into mechanisms of ES cell self-renewal and pluripotency.

## Results

### Co-localization of H4K20me3 with H3K4me3 and RNAPII in ES cells

**Fig. 1:**
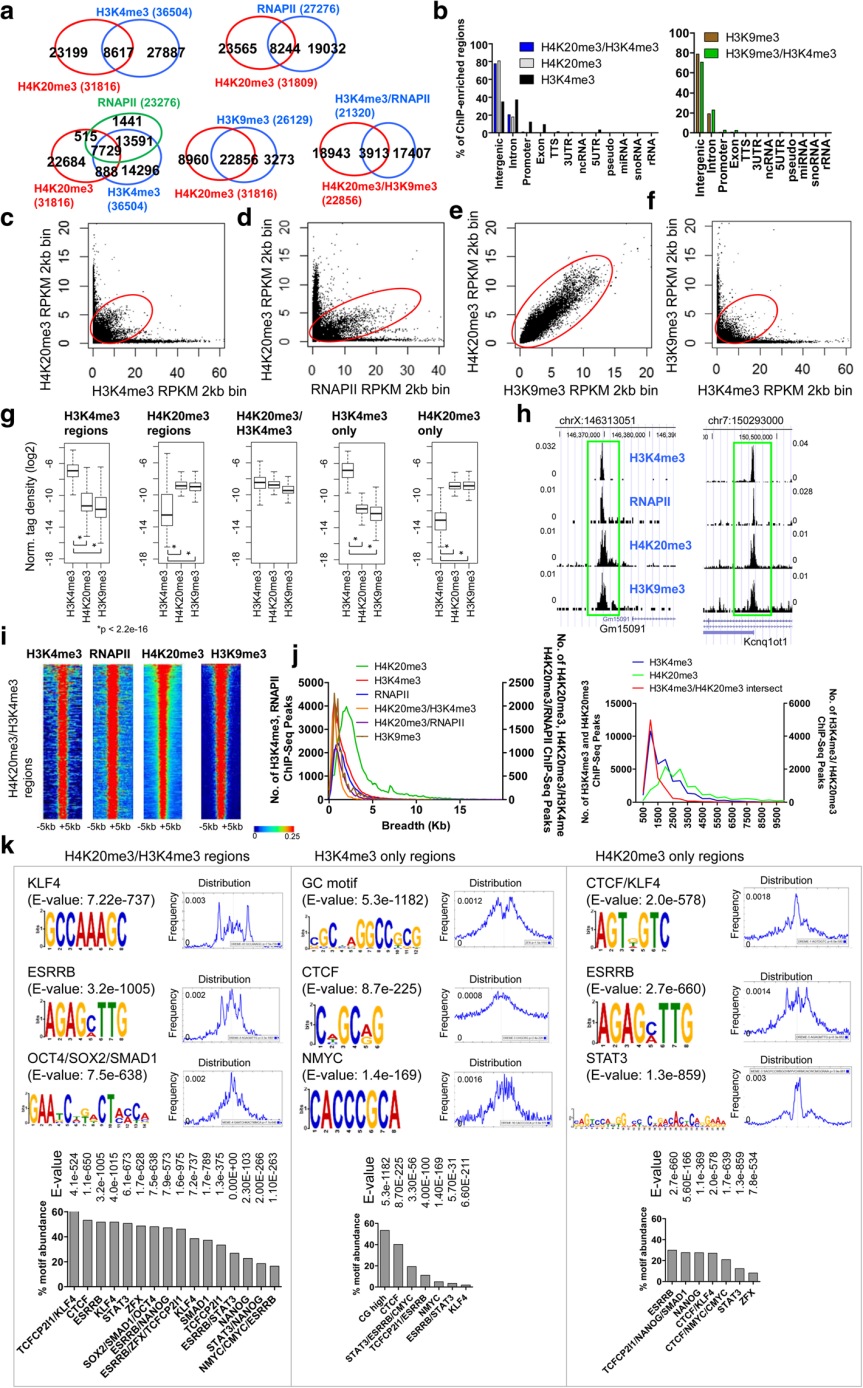
Co-localization of H4K20me3 with H3K4me3 and RNAPII in ES cells. **a** Venn diagrams showing overlap between H4K20me3 and H3K4me3, H4K20me3 and RNAPII, H4K20me3/H3K4me3/RNAPII, H4K20me3 and H3K9me3, and H3K4me3/RNAPII/H4K20me3/H3K9me3 co-occupied regions. **b** Annotation of H4K20me3 and H3K4me3, and H3K9me3 and H3K4me3 co-occupied regions using HOMER software [40]. Scatter plots of (**c**) H4K20me3 and H3K4me3 densities, **d** H4K20me3 and RNAPII, **e** H4K20me3 and H3K9me3 densities, and (**f**) H3K9me3 and H3K4me3 densities (RPKM) at 2 kb genomic bin intervals. **g** Density of H3K4me3 and H4K20me3 at H3K4me3 peaks (left panel), H4K20me3 peaks (middle panel), H4K20me3/H3K4me3 intersecting regions, H3K4me3 only peaks, or H4K20me3 only peaks (right panel). **h** UCSC browser view of H4K20me3, H3K9me3, H3K4me3, and RNAPII co-occupancy in ES cells. **i** Heat maps of H3K4me3, RNAPII, H4K20me3, and H3K9me3 densities at H4K20me3/H3K4me3 marked regions. Rows were sorted by the level of H4K20me3 at H4K20me3/H3K4me3 regions. **j** Distribution of H4K20me3, H3K9me3, H3K4me3 and H4K20me3/H3K4me3 co-occupied ChIP-Seq peaks in ES cells. **k** Enriched DNA binding motifs from Chen et al. [41] in H4K20me3/H3K4me3, H3K4me3-only, and H4K20me3-only co-occupied regions identified using MEME-ChIP software [42]. The percent abundance of motifs is shown below the sequence logo

To investigate whether H4K20me3 is enriched at genes with activating histone modifications in ES cells, we compared ChIP-Seq regions occupied by H4K20me3 (GSE94086) [17] and H3K4me3 (GSE53093) [4], and H4K20me3 and RNAPII (GSE94739) [18], and found that 27% of H4K20me3 occupied regions contain H3K4me3 marks (Fig. 1a, top left), and 26% were bound by RNAPII (Fig. 1a, top right). Moreover, 94% of H4K20me3/RNAPII regions (7729/8224) intersect with H4K20me3/H3K4me3 regions (Fig. 1a, bottom left). We then compared the overlap between H4K20me3 and H3K9me3 (GSE94086) [17] ChIP-Seq regions, and found that 72% of H4K20me3 overlap with H3K9me3 peaks, and 12% of H4K20me3 peaks were co-occupied with H3K9me3, H3K4me3, and RNAPII (Fig. 1a, bottom right).

Annotation of H4K20me3/H3K4me3 enriched regions showed that they mainly reside in intergenic (78%) and intron (21%) regions (Fig. 1b). Likewise, H3K9me3/H3K4me3 regions are mainly located in intergenic (70%) and intron (23%) regions (Fig. 1b, right). Co-occupancy of H4K20me3/H3K4me3 and H4K20me3/RNAPII is also visible at a subset of regions upon inspection of H4K20me3 and H3K4me3 densities (Fig. 1c), and H4K20me3 and RNAPII densities (Fig. 1d) at 2 kb genomic intervals. While H4K20me3 and H3K4me3 co-occupy a subset of regions, H4K20me3 and H3K9me3 (GSE94086) [17] levels highly overlap at most regions (Fig. 1e). Co-occupancy of H3K9me3/H3K4me3 was also visible at a subset of regions following an evaluation of H3K9me3 and H3K4me3 at 2 kb genomic intervals (Fig. 1f). Interestingly, while H4K20me3 and H3K9me3 levels are overall lower at all H3K4me3 regions, or at H3K4me3 only regions (Fig. 1g), and H3K4me3 levels are overall lower at all H4K20me3 regions, or at H4K20me3 only regions (Fig. 1g), H4K20me3, H3K9me3 and H3K4me3 levels are largely similar at H4K20me3/H3K4me3 co-occupied regions (Fig. 1g, middle). These results also show that H3K4me3 levels are slightly higher at all H3K4me3 regions relative to H4K20me3/H3K4me3 co-occupied regions, but lower at H4K20me3 regions (Fig. 1g). In addition, H4K20me3 and H3K9me3 levels are similar at all H4K20me3 regions and at H4K20me3/H3K4me3 regions, but lower at all H3K4me3 regions (Fig. 1g). Inspection of custom tracks on the UCSC genome browser revealed enrichment of H3K4me3, RNAPII, H4K20me3, and H3K9me3 at several regions (Fig. 1h). Moreover, we observed enrichment of H4K20me3, H3K9me3, H3K4me3, and RNAPII at H4K20me3/H3K4me3 co-occupied regions by heat maps (Fig. 1i). The breadth of H4K20me3/H3K4me3 and H4K20me3/RNAPII domains was similar to H3K4me3 domains, where the majority were 1-5 kb in length (Fig. 1j), while H4K20me3 domains were broader (1-15 kb) (Fig. 1j, left). Moreover, our results show that > 85% of H4K20me3/H3K4me3 regions overlapped by at least 1 kb (Fig. 1j, right).

In addition, motif analysis of H4K20me3/H3K4me3, H3K4me3 only, and H4K20me3 only marked regions revealed greater enrichment of pluripotency-specific transcription factors (TF) including ESRRB, KLF4, SOX2, OCT4, SMAD1, STAT3, and ZFX, and the chromatin insulator CTCF, in H4K20me3/H3K4me3 marked regions relative to H3K4me3 only or H4K20me3 only regions (Fig. 1k). In addition, H3K4me3 only regions were highly enriched at GC-rich sequences (Fig. 1k). Collectively, these results suggest that H4K20me3/H3K4me3 co-occupies DNA sequences regulated by the core transcriptional regulatory circuitry of mouse ES cells.

### Global sequential ChIP confirms co-occupancy of H4K20me3/H3K4me3

**Fig. 2:**
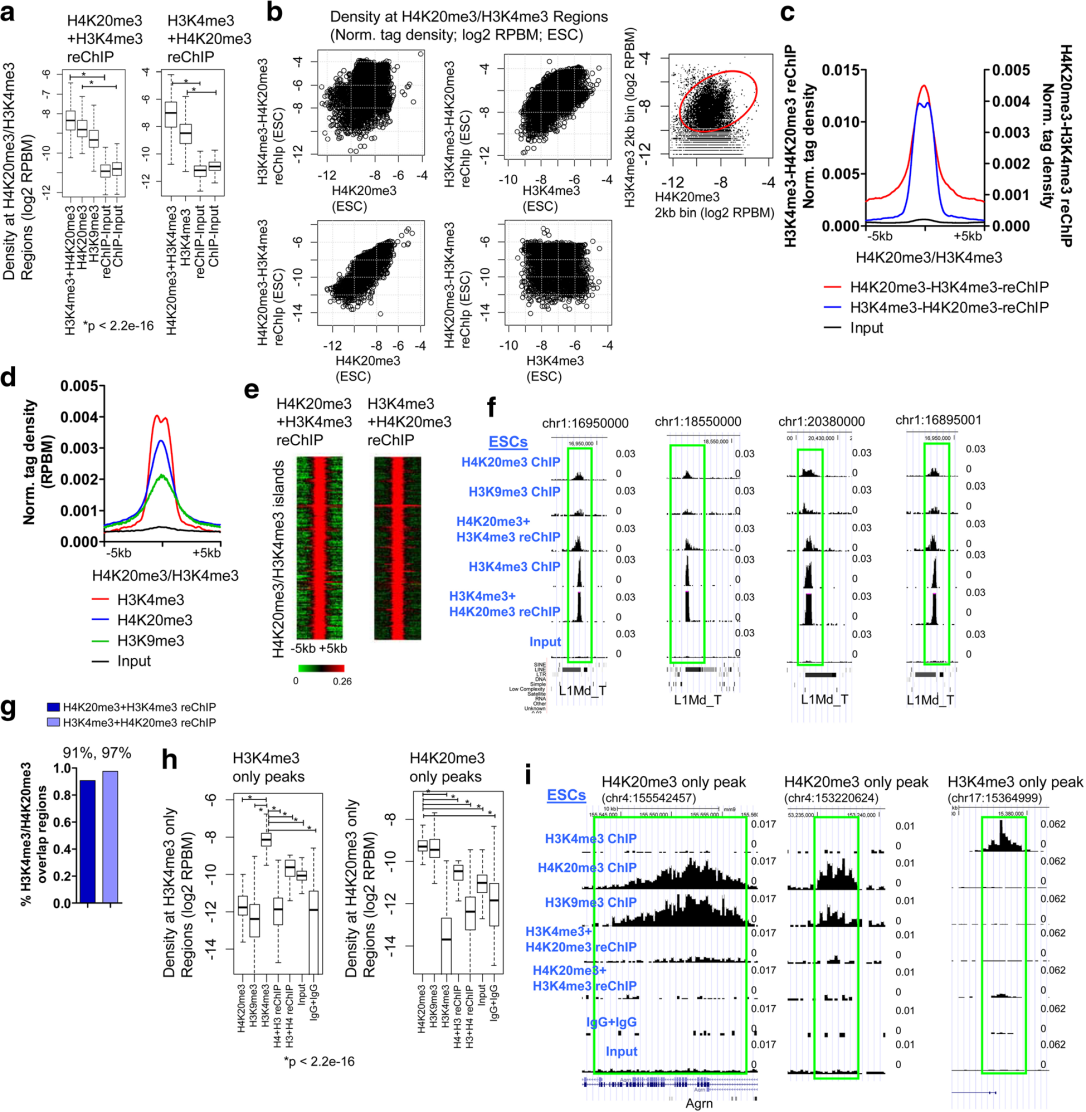
Re-ChIP-Seq Validation of H4K20me3 and H3K4me3 co-occupancy in ES cells. **a** Boxplot of density of H3K4me3 + H4K20me3 reChIP (left) or H4K20me3 + H3K4me3 reChIP (right) at H4K20me3/H3K4me3 intersecting islands. **b** Scatter plot of H3K4me3 + H4K20me3 reChIP and H4K20me3 density (top left), H3K4me3 + H4K20me3 reChIP and H3K4me3 density (top middle), H4K20me3 + H3K4me3 reChIP and H4K20me3 density (bottom left), and H4K20me3 + H3K4me3 and H3K4me3 density (bottom right) at H4K20me3/H3K4me3 intersecting islands. Scatter plot of H4K20me3 and H3K4me3 densities (RPKM) at 2 kb genomic bin intervals, which intersect H4K20me3/H3K4me3 ChIP bivalent regions (top right). **c** Average density profiles of H3K4me3 + H4K20me3 and H4K20me3 + H3K4me3 at H4K20me3/H3K4me3 intersecting islands, and (**d**) H3K4me3, H4K20me3, and H3K9me3 at H4K20me3/H3K4me3 intersecting regions. **e** Heatmaps of density of H3K4me3 + H4K20me3 and H4K20me3 + H3K4me3 at H4K20me3/H3K4me3 intersecting islands. Rows were sorted by chromosome number and position. **f** Browser view of H3K4me3 + H4K20me3 and H4K20me3 + H3K4me3 reChIP-Seq at individual L1/ L1Md_T repeat subfamily elements. **g** Percent of H4K20me3/H3K4me3 regions that overlap with H4K20me3 + H3K4me3 reChIP and H3K4me3 + H4K20me3 peaks. **h** Boxplot of ChIP-Seq and reChIP-Seq densities at regions marked by only H3K4me3 (left) or H4K20me3 (right). **i** Browser view of ChIP-Seq and reChIP-Seq signals at regions marked by H4K20me3/H3K9me3 only (left, middle) or H3K4me3 only (right)

To determine whether both H4K20me3 and H3K4me3 are simultaneously present at the same genomic regions, we performed Re-ChIP, also termed sequential ChIP [3], by immunoprecipitating ES cell chromatin first with an H4K20me3 or an H3K4me3 antibody, and second with an H3K4me3 or H4K20me3 antibody, respectively. We then performed reChIP-Seq and evaluated the density of H4K20me3 + H3K4me3 reChIP or H3K4me3 + H4K20me3 reChIP at H4K20me3/H3K4me3 co-occupied regions. H4K20me3 + H3K4me3 reChIP or H3K4me3 + H4K20me3 reChIP levels were significantly elevated at H4K20me3/H3K4me3 co-marked regions relative to the control (Input) (Fig. 2a-b), demonstrating that a subset of H4K20me3 marked regions contain H3K4me3 marks. We also found that H4K20me3 + H3K4me3 reChIP or H3K4me3 + H4K20me3 reChIP levels were comparable to H4K20me3 or H3K4me3 ChIP-Seq levels (Fig. 2b). An evaluation of H4K20me3 and H3K4me3 densities at 2 kb genomic intervals, which intersect H4K20me3/H3K4me3 regions, showed that H3K4me3 and H4K20me3 reChIP-Seq levels are relatively similar to ChIP-Seq levels (Fig. 2b, right). Average profiles and heatmaps also reveal enrichment of H4K20me3 + H3K4me3 reChIP, H3K4me3 + H4K20me3 reChIP, H3K4me3, H4K20me3, and H3K9me3 at H4K20me3/H3K4me3 co-marked regions (Fig. 2c-e). Moreover, browser views showed elevated levels of H4K20me3 + H3K4me3 and H3K4me3 + H4K20me3 at H4K20me3/H3K4me3 regions containing individual L1Md_T repeat subfamily elements (Fig. 2f). We also evaluated the fraction of H4K20me3/H3K4me3 domains (shown in Fig. 1a) which overlap with H4K20me3 + H3K4me3 reChIP and H3K4me3 + H4K20me3 reChIP peaks. Our results demonstrate that 91 and 97% of H4K20me3/H3K4me3 co-occupied regions overlap with H4K20me3 + H3K4me3 reChIP and H3K4me3 + H4K20me3 peaks, respectively (Fig. 2g).

To further evaluate the specificity of the reChIP signals, we investigated H4K20me3 + H3K4me3 reChIP and H3K4me3 + H4K20me3 reChIP signals at regions marked by only H3K4me3 or H4K20me3 in ES cells. Using this approach, we observed enrichment of H3K4me3 ChIP-Seq signals at H3K4me3 only regions, but did not observe significant enrichment of H4K20me3 + H3K4me3 reChIP and H3K4me3 + H4K20me3 reChIP signals at these regions (Fig. 2h, left). In addition, we observed enrichment of H4K20me3 ChIP-Seq signals at H4K20me3 only regions, but did not observe significant enrichment of H4K20me3 + H3K4me3 reChIP and H3K4me3 + H4K20me3 reChIP signals at these regions (Fig. 2h, right). Custom UCSC browser views also showed elevated levels of H4K20me3 and H3K9me3 at regions containing only H4K20me3 peaks, and enrichment of H3K4me3 at regions containing only H3K4me3 peaks (Fig. 2i). Combined, these results suggest that a subset of H4K20me3 marked regions contain H3K4me3 marks.

### H4K20me3/H3K4me3 bivalently marked genes are active in ES cells

**Fig. 3:**
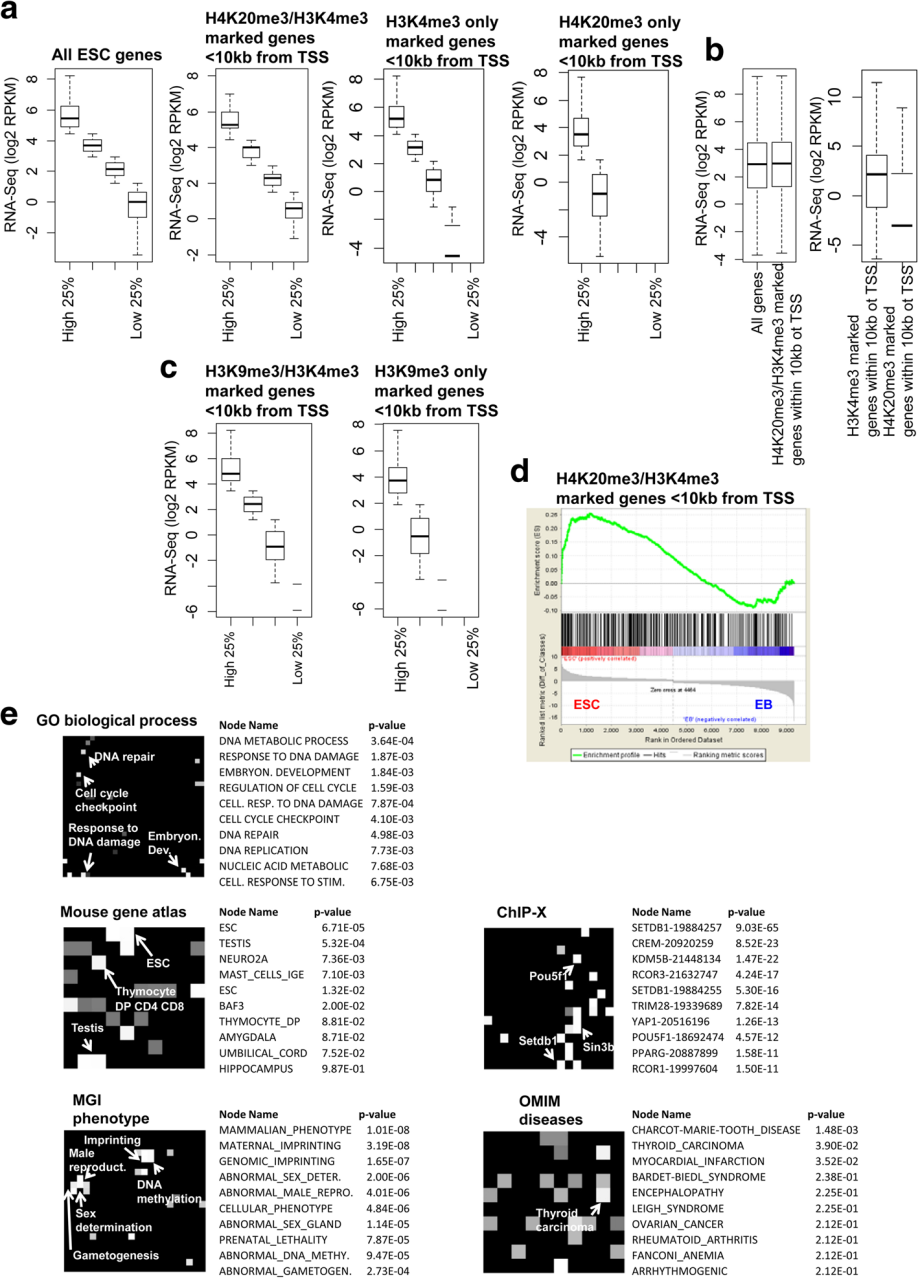
Expression and network analysis of H4K20me3/H3K4me3 associated genes. **a** Boxplot of RNA-Seq expression data for all genes in ES cells (left), genes containing H4K20me3/H3K4me3 marks, genes with only H3K4me3 marks, and genes with only H4K20me3 marks within 10 kb of TSS. All genes, or genes containing H4K20me3/H3K4me3, H3K4me3-only, or H4K20me3-only were divided into quartiles based on their expression in ES cells. **b** Boxplot of RNA-Seq expression data for all genes and genes containing H4K20me3/H3K4me3 marks within 10 kb of TSS (right). **c** Boxplot of RNA-Seq expression data for genes containing H3K9me3/H3K4me3 marks and genes with only H3K9me3 marks within 10 kb of TSS (middle). **d** Gene set enrichment analysis (GSEA) of H4K20me3/H3K4me3 co-marked genes in ES cells relative to differentiated embryoid bodies (EBs). **e** Network2Canvas analyses of genes containing H4K20me3/H3K4me3 marks within 10 kb of TSS. Each node (square) represents a gene list (H4K20me3/H3K4me3 co-occupied genes associated with a gene-set library (Gene ontology (GO) biological process, mouse gene atlas, MGI phenotype, ChIP-X or OMIM diseases). The brightness (white) of each node is determined by its *P*-value

To investigate whether H4K20me3/H3K4me3 bivalent marks are positively or negatively associated with gene activity we evaluated the expression state of genes containing H4K20me3/H3K4me3 marks within 10 kb of their transcriptional start site (TSS) using RNA-Seq data from ES cells (GSE47124) [19]. These results demonstrate that most genes containing H4K20me3/H3K4me3 marks are expressed in ES cells (RPKM> 1) (Fig. 3a). In addition, genes containing H4K20me3/H3K4me3 marks exhibit higher expression relative to genes with H4K20me3 only (Fig. 3a, right). Moreover, genes containing H4K20me3/H3K4me3 have a similar distribution of expression relative to all genes in ES cells, or genes marked with H3K4me3 only, while genes marked with H4K20me3 only exhibit decreased expression (Fig. 3b). Likewise, we found that most genes containing H3K9me3/H3K4me3 marks are expressed in ES cells (RPKM> 1) (Fig. 3c). Moreover, genes containing H3K9me3/H3K4me3 marks exhibit higher expression relative to genes with H3K9me3 only (Fig. 3c, right). To further evaluate the expression state of genes containing H4K20me3/H3K4me3 marks within 10 kb of their TSS we compared these genes to expression data from undifferentiated ES cells and day 14 embryoid body (EB) differentiated ES cells [19] using gene set enrichment analysis (GSEA) [20]. These results show that expression of genes containing H4K20me3/H3K4me3 marks within 10 kb of their TSS is enriched in undifferentiated ES cells relative to differentiated EBs (Fig. 3d). Further expression analysis of H4K20me3/H3K4me3 co-marked genes using Network2canvas [21] revealed that H4K20me3/H3K4me3 marked genes are highly expressed in ES cells, testis, and thymocytes (Fig. 3e). Moreover, gene ontology (GO) terms enriched in H4K20me3/H3K4me3 marked genes include embryonic development, cell cycle, DNA repair, and response to DNA damage (Fig. 3e). Further annotation revealed that genes nearby H4K20me3/H3K4me3 marks are bound by OCT4, SETDB1, and SIN3B, are involved in DNA methylation, imprinting, gametogenesis, and sex determination, and are associated with thyroid carcinoma (Fig. 3e). These results suggest that H4K20me3/H3K4me3 bivalent marks are associated with genes involved in multiple cellular processes.

### H4K20me3/H3K4me3 marks transcriptionally dynamic genes in ES cells

**Fig. 4:**
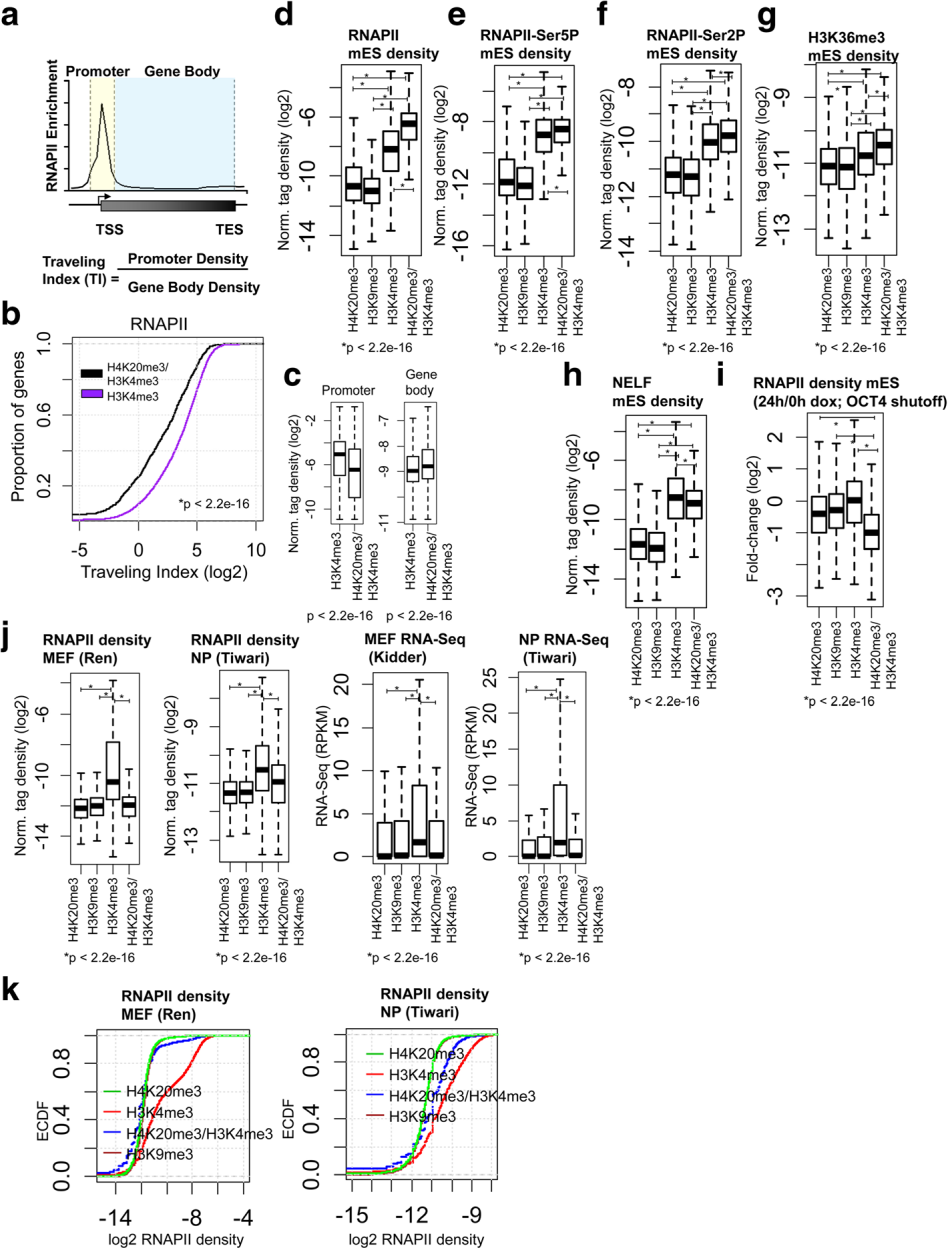
H4K20me3/H3K4me3 marks transcriptionally dynamic genes in ES cells. **a** Schematic describing the calculation used to determine the traveling index (TI) at RNAPII marked genes in ES cells. The promoter bin is defined as a 1 kb window around the TSS of genes marked by RNAPII, while the transcribed region (gene body) is defined as the region extending to the TES. The TI is calculated from the ratio of the density of RNAPII in the promoter bin to the density of RNAPII in the gene body bin. **b** Empirical cumulative distribution for the TI of RNAPII across H4K20me3/H3K4me3 (black) and H3K4me3 (purple) marked genes in ES cells. Y-axis shows the percentage of genes that exhibit a TI less than the value specified by the x-axis. A line shifted to the left means a systematic decrease in the traveling index. *p*-value < 2.2e-16 (Kolmogorov-Smirnov test). Note the decreased TI for genes marked by H4K20me3/H3K4me3. **c** Boxplot of RNAPII density in promoter (left) and gene body (right) regions at H4K20me3/H3K4me3 and H3K4me3 regions. **d-h** Boxplots of (**d**) RNAPII, **e** RNAPII-Ser5P, **f** RNAPII-Ser2P, **g** H3K36me3, and (**h**) NELF densities at H4K20me3/H3K4me3, H3K4me3, H4K20me3, and H3K9me3 regions in ES cells. **i-j** Boxplots of RNAPII density (**i**) 24 h following OCT4 shutdown in ZHTBc4 ES cells and in (**j**) mouse fibroblasts (left) and neural progenitors (right). **k** Empirical cumulative distribution function (ECDF) for RNAPII density in mouse fibroblasts (left) and neural progenitors

To investigate whether dual marking of genes by the repressive H4K20me3 and activating H3K4me3 histone modifications is a regulatory mechanism that controls RNA polymerase II (RNAPII) recruitment or promoter-proximal pausing, we evaluated RNAPII occupancy (GSE94739) [18] at genes marked by H4K20me3/H3K4me3 relative to genes marked by H3K4me3. To evaluate the level of pausing at genes containing H4K20me3/H3K4me3 or H3K4me3, we quantified the relative ratio of RNAPII in promoter to that in gene body regions, which has been termed the ‘traveling index’ (TI) [18] (Fig. 4a). Genes with a higher TI reflect greater enrichment of RNAPII binding at promoter regions relative to gene body regions. For genes where the rate of promoter clearance is similar to the rate of initiation, the TI is close to 1 [22, 23], while genes with a TI greater than 1 exhibit promoter clearance of RNAPII at a rate lower than the initiation rate [23]. It was previously found that 91% of genes in ES cells exhibit a RNAPII TI greater than 2, demonstrating that RNAPII density is greater in proximal-promoter regions relative to gene body regions [23]. Using this calculation, we observed a decrease in the TI for RNAPII at genes marked by H4K20me3/H3K4me3 (black) relative to genes marked by H3K4me3 (purple) in ES cells (Fig. 4b): We observed 84% of genes containing H3K4me3 with a RNAPII TI greater than 2, but only 65% of H4K20me3/H3K4me3 bivalently marked genes had a RNAPII TI greater than 2 (Fig. 4b). Our results also show that genes marked by H4K20me3/H3K4me3 have higher RNAPII binding in gene body regions relative to genes marked by H3K4me3 (Fig. 4c, right). In contrast, RNAPII binding was lower at promoter regions of genes containing H4K20me3/H3K4me3 relative to genes marked by H3K4me3 (Fig. 4c, left). Interestingly, RNAPII binding [18] was higher at H4K20me3/H3K4me3 regions relative to H3K4me3, H4K20me3, or H3K9me3 regions (Fig. 4d, left), suggesting that the genes marked by H4K20me3/H3K4me3 may exhibit an altered rate of transcriptional elongation. These findings also suggest that a possible function of H4K20me3/H3K4me3 is to contribute to pause release, where we observe that most genes associated with H4K20me3/H3K4me3 are actively transcribed in ES cells. While we observed a correlation between genes marked by H4K20me3/H3K4me3 and elevated RNAPII binding, further studies will be required to determine whether dual marking with H4K20me3/H3K4me3 regulates transcriptional elongation.

We also evaluated whether H4K20me3/H3K4me3 marks regulate the rate-limiting step of initiation after RNAPII is recruited to promoters and subsequently modified on the c-terminal domain (CTD) to Ser5P on the large subunit [24]. An evaluation of ChIP-Seq data [18] revealed higher binding of RNAPII-Ser5P at H4K20me3/H3K4me3 regions relative to H3K4me3, H4K20me3, or H3K9me3 regions (Fig. 4e). Likewise, to investigate whether H4K20me3/H3K4me3 marks are correlated with RNAPII elongation rates, we evaluated RNAPII-Ser2P binding at H4K20me3/H3K4me3 regions relative to H3K4me3 or H4K20me3 regions. Analysis of ChIP-Seq data [18] also revealed higher binding of RNAPII-Ser2P at H4K20me3/H3K4me3 regions relative to H3K4me3, H4K20me3, or H3K9me3 regions (Fig. 4f). In addition, H3K36me3, which is associated with active transcriptional elongation, was also higher at H4K20me3/H3K4me3 regions relative to H3K4me3, H4K20me3, or H3K9me3 regions (Fig. 4g).

Because we observed altered RNAPII binding at H4K20me3/H3K4me3 regions relative to H3K4me3 or H4K20me3 regions, indicative of decreased RNAPII pausing, we investigated whether negative elongation factor (NELF), which pauses RNAPII just downstream of the transcription start site (TSS) [25, 26], also exhibits altered binding at these regions. Indeed, an analysis of ChIP-Seq data (GSE20530) demonstrated that NELF binding in ES cells is lower at H4K20me3/H3K4me3 co-marked regions relative to H3K4me3 regions (Fig. 4h), indicating that H4K20me3/H3K4me3 regions may have decreased pausing.

If H4K20me3/H3K4me3 marks genes that are actively transcribed in ES cells, it is possible that these histone modifications may poise ESC-enriched genes in a bipotential state that is amenable to rapid deactivation of RNAPII binding during differentiation in the presence of intrinsic or external signals. To test this possibility, we analyzed public data where a doxycycline-inducible OCT4 shutdown mES cell line [27] was utilized to monitor RNAPII levels by ChIP-Seq before and after OCT4 shutdown [23]. Following 24 h after treatment with doxycycline to downregulate OCT4 levels, RNAPII binding was reduced more at H4K20me3/H3K4me3 regions relative to H3K4me3, H4K20me3, or H3K9me3 regions (Fig. 4i), suggesting that H4K20me3/H3K4me3 marked genes are poised for rapid deactivation of RNAPII binding upon downregulation of OCT4. Likewise, to investigate whether H4K20me3/H3K4me3 poises genes for deactivation of RNAPII binding during lineage-specific differentiation, we evaluated RNAPII binding using public ChIP-Seq data from mouse fibroblasts (MEFs) (GSE71507) and neural progenitor cells (NP) (GSE89573) [28]. Results from these analyses demonstrate that RNAPII binding was significantly lower at H4K20me3/H3K4me3 regions relative to H3K4me3 regions (Fig. 4j-k, left). We also evaluated the RNA expression levels of genes co-marked with H4K20me3/H3K4me3 relative to H4K20me3, H3K9me3, or H3K4me3 regions in MEFs (see methods) and NPs (GSE89574) [28]. Our findings demonstrate that genes co-marked by H4K20me3/H3K4me3 in ES cells is significantly lower relative to genes marked by H3K4me3 (Fig. 4j, right). Overall, these results demonstrate that dual marking of active genes by H4K20me3/H3K4me3 in ES cells may facilitate rapid deactivation during differentiation.

### H4K20me3 co-localizes with H3K36me3 in gene body regions

**Fig. 5:**
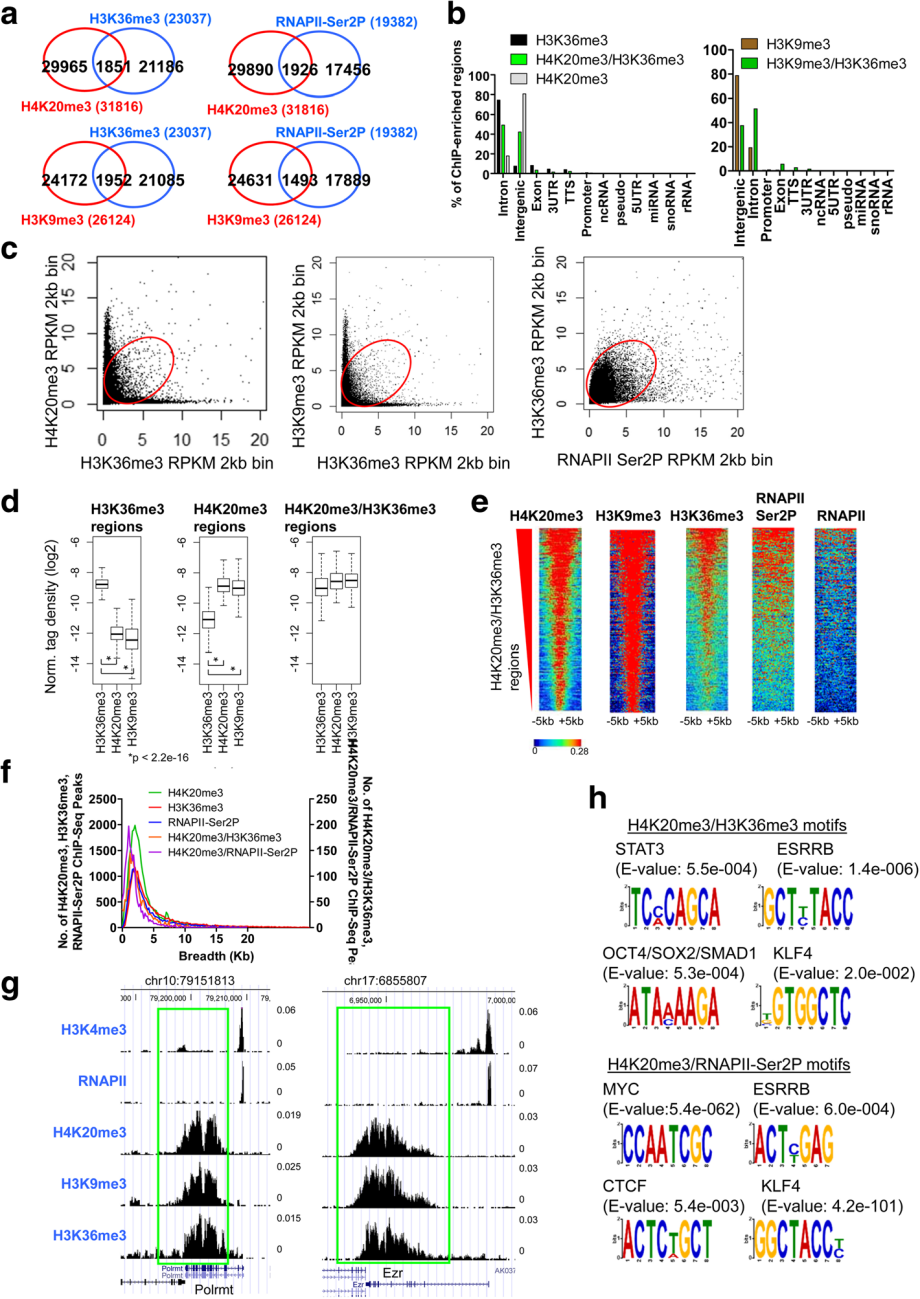
H4K20me3 co-localizes with H3K36me3 in gene body regions of a subset of active genes in ES cells. **a** Venn diagram showing overlap between H4K20me3 and H3K36me3, H4K20me3 and RNAPII-Ser2P, H3K9me3 and H3K36me3, and H3K9me3 and RNAPII-Ser2P co-occupied regions. **b** Annotation of H4K20me3 and H3K36me3, and H3K9me3 and H3K36me3 co-occupied regions using HOMER software. Scatter plot of (**c**) H4K20me3 and H3K36me3 densities (RPKM), and H3K9me3 and H3K36me3 densities at 2 kb genomic bin intervals. **d** Density of H3K36me3, H4K20me3, and H3K9me3 at H3K36me3 peaks (left panel), H4K20me3 peaks (middle panel), and H4K20me3/H3K36me3 intersecting regions (right panel). **e** Heat maps of H4K20me3, H3K9me3, H3K36me3, RNAPII-Ser2P, and RNAPII densities at H4K20me3/H3K36me3 marked regions. Rows were sorted by the level of H4K20me3 at H4K20me3/H3K36me3 regions. **f** Distribution of H4K20me3, H3K36me3, RNAPII-Ser2P, H4K20me3/H3K36me3, and H4K20me3/RNAPII-Ser2P co-occupied ChIP-Seq peaks in ES cells. **g** UCSC browser view of H4K20me3, H3K9me3, and H3K36me3 co-occupancy in ES cells. **h** Enriched DNA binding motifs in H4K20me3/H3K36me3 co-occupied regions identified using MEME-ChIP software

Because we observed co-occupancy of the repressive histone modification, H4K20me3, with the activating histone modification, H3K4me3, at a subset of gene promoters in ES cells, we evaluated whether H4K20me3 co-localizes with another activating histone modification, histone 3, lysine 36 trimethylation (H3K36me3), in gene body regions of active genes in ES cells. To this end, we first interrogated H3K36me3 localization in ES cells using ChIP-Seq (see methods). Interestingly, we found that 8% of H3K36me3 regions were also marked by H4K20me3 (Fig. 5a, left), and 10% were co-occupied by elongating RNA polymerase II (RNAPII Ser2P) (Fig. 5a, right). RNAPII is phosphorylated on the serine 2 residue (Ser2) of the C-terminal domain (CTD) of the large subunit during transcriptional elongation [18, 24]. We also compared the overlap between H3K9me3 and H3K36me3 ChIP-Seq regions, and found that 8% of H3K36me3 peaks were co-occupied with H3K9me3 (Fig. 5a, bottom), and 8% were occupied by RNAPII Ser2P (Fig. 5a, bottom right). In addition, 41% of H4K20me3/H3K36me3 co-occupied peaks were occupied with H3K4me3, while 9% of H4K20me3/H3K4me3 regions were co-occupied with H3K36me3. Annotation of H4K20me3/H3K36me3 and H3K9me3/H3K36me3 co-occupied regions revealed that they are predominantly localized in intronic and intergenic regions (Fig. 5b). While genome-wide co-occupancy of H3K36me3/RNAPII-Ser2P is visible when evaluating H3K36me3 and RNAPII densities at 2 kb genomic intervals, only a subset of H4K20me3/H3K36me3 marked regions is evident when evaluating H4K20me3 and H3K36me3 densities at 2 kb genomic intervals (Fig. 5c). In addition, a subset of H3K9me3/H3K36me3 regions are evident when evaluating H3K9me3 and H3K36me3 densities at 2 kb genomic intervals (Fig. 5c). Moreover, while H4K20me3 and H3K9me3 levels are overall lower at all H3K36me3 regions (Fig. 5d, left), and H3K36me3 levels are overall lower at all H4K20me3 regions (Fig. 5d, middle), H4K20me3, H3K9me3, and H3K36me3 levels are relatively similar at H4K20me3/H3K36me3 co-marked regions (Fig. 5d, right). Heat maps also demonstrate co-enrichment of H4K20me3, H3K36me3, and H3K9me3 at a subset of regions (Fig. 5e). Moreover, we observed enrichment of elongating RNAPII (Ser2P) at regions co-occupied by H4K20me3/H3K36me3 (Fig. 5e). However, we did not observe enrichment of total RNAPII at regions co-occupied by H4K20me3/H3K36me3 (Fig. 5e). Combined, these results suggest that H4K20me3 co-localizes with H3K36me3 and RNAPII-Ser2P in gene body regions of a subset of genes.

In addition, the breadth of H4K20me3/H3K36me3 and H4K20me3/RNAPII-Ser2P domains was similar to H3K36me3 and H4K20me3 domains (Fig. 5f). H4K20me3, H3K9me3 and H3K36me3 co-localization is visible at several regions in UCSC genome browser views (Fig. 5g). To gain further insight into sequences marked by H4K20me3/H3K36me3 we performed motif analysis of H4K20me3/H3K36me3 marked regions, and identified enrichment of pluripotency-regulators such as STAT3, ESRRB, KLF4, OCT4, SOX2, and SMAD1 (Fig. 5h, left). Moreover, motif analysis of H4K20me3/RNAPII-Ser2P co-occupied regions demonstrated enrichment of MYC, ESRRB, CTCF, and KLF4 transcriptional regulators (Fig. 5h, right). Altogether, these results suggest that H4K20me3/H3K36me3 and H4K20me3/RNAPII-Ser2P co-occupied DNA sequences may be regulated by pluripotency-regulators in ES cells.

### Enrichment of repetitive DNA elements in H4K20me3-marked bivalent domains

**Fig. 6:**
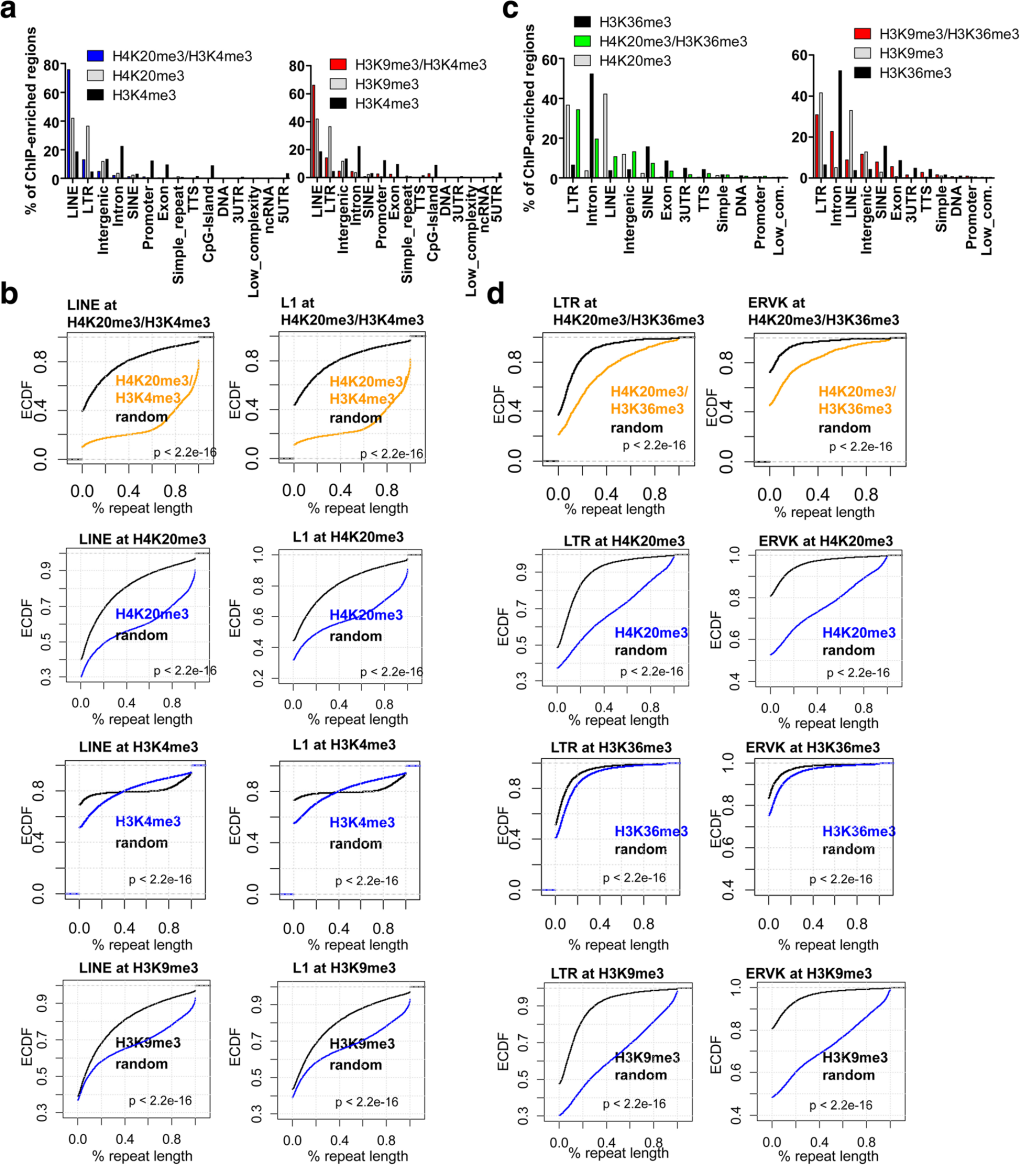
H4K20me3 and H3K4me3 occupy L1 and ERVK family repetitive elements in ES cells. **a** Annotation of H4K20me3/H3K4me3, H4K20me3, and H3K4me3 enriched regions (left) and H3K9me3/H3K4me3, H3K9me3, and H3K4me3 regions (right) in ES cells using HOMER software. **b** LINE class and L1 family of repetitive DNA sequences are enriched in H4K20me3/H3K4me3 co-occupied regions, H4K20me3 regions, and H3K9me3 regions. Empirical cumulative distribution plots for the percent coverage of LINE repeats (left) or the L1 repeat family member (right) across H4K20me3/H3K4me3 co-marked regions (orange), H4K20me3 regions (blue), H3K9me3 regions (blue), or H3K4me3 regions (blue) relative to their respective random genomic regions (black). Y-axis shows the percentage of genes with a percent repeat length less than the value specified by the x-axis. A line shifted to the right means a systematic increase in the percent coverage of a repeat element in ChIP-Seq peaks relative to random genomic sequences. P-value for all < 2.2e-16 (Kolmogorov-Smirnov test). **c** Annotation of H4K20me3/H3K36me3, H4K20me3, and H3K36me3 enriched regions (left) and H3K9me3/H3K36me3, H3K9me3, and H3K36me3 regions (right) in ES cells using HOMER software. **d** LTR repetitive DNA sequence classes are enriched in H4K20me3/H3K36me3 co-occupied regions. Empirical cumulative distribution for the percent coverage of LTR repeats (left) or the ERVK repeat family member (right) across H4K20me3/H3K36me3 co-marked regions (orange), H4K20me3 regions (blue), H3K9me3 regions (blue), or H3K4me3 regions (blue) relative to their respective random genomic regions (black). Y-axis shows the percentage of genes with a percent repeat length less than the value specified by the x-axis. A line shifted to the right means a systematic increase in the percent coverage of a repeat element in ChIP-Seq peaks relative to random genomic sequences. P-value for all < 2.2e-16 (Kolmogorov-Smirnov test)

Because H4K20me3 has previously been shown to be enriched at repetitive DNA sequences [2, 17], we investigated whether DNA repeats are enriched in sequences marked by H4K20me3/H3K4me3 and H4K20me3/H3K36me3 bivalent domains. By annotating H4K20me3/H3K4me3 using HOMER software, we found that H4K20me3/H3K4me3 co-marked regions are enriched with long interspersed elements (LINE) (Fig. 6a). Our results show that 76% of H4K20me3/H3K4me3 co-marked regions are enriched with LINE elements, while 42% of H4K20me3 regions contain LINE elements (Fig. 6a). However, only 18% of H3K4me3 marked regions contain LINE elements. In contrast, while LTR elements are enriched in H4K20me3 and H3K9me3 regions [17] (Fig. 6a), LTR elements are not significantly enriched in H4K20me3/H3K4me3 or H3K9me3/H3K4me3 co-marked regions or regions occupied by H3K4me3. In addition, 66% of H3K9me3/H3K4me3 co-marked regions are enriched with LINE elements, while 42% of H3K9me3 regions contain LINE lements (Fig. 6a, right).

We then calculated the percent coverage of H4K20me3/H3K4me3 peaks that overlap LINE repeat elements, and found that H4K20me3/H3K4me3 co-marked regions are enriched with LINE repeats relative to random genomic regions (Fig. 6b, left). We also observed enrichment of the L1 (LINE class) family repetitive elements in H4K20me3/H3K4me3 regions (Fig. 6b, right). In addition, we observed enrichment of LINE and L1 repeats in all H4K20me3 and H3K9me3 regions [17] (Fig. 6b), but decreased enrichment of LINE and L1 repeats in all H3K4me3 regions [17] (Fig. 6b). Likewise, we annotated H4K20me3/H3K36me3 regions using HOMER software, and observed enrichment of long-terminal repeat (LTR) elements in H4K20me3/H3K36me3 regions (Fig. 6c). Our results show that 34% of H4K20me3/H3K36me3 regions and 37% of H4K20me3 regions contain LTR elements (Fig. 6c). However, only 6% of H3K36me3 marked regions contain LTR elements. In addition, 31% of H3K9me3/H3K36me3 regions and 42% of H3K9me3 regions contain LTR elements (Fig. 6c, right).

Next, we calculated the percent coverage of H4K20me3/H3K36me3 peaks that overlap LTR repeat elements, and found that H4K20me3/H3K36me3 co-marked regions are enriched with LTR repeats relative to random genomic sequences (Fig. 6d, left). We also observed enrichment of the ERVK (LTR class) family repetitive elements in H4K20me3/H3K36me3 regions (Fig. 6d, right). While LTR and ERVK repeats were enriched in H4K20me3 and H3K9me3 regions (Fig. 6d), enrichment of LTR and ERVK repeats was lower in H3K36me3 regions (Fig. 6d, bottom). Altogether, these results suggest that co-localization of H4K20me3/H3K4me3 occurs mainly in intergenic regions, which are also enriched with LINE elements, while co-localization of H4K20me3/H3K36me3 occurs predominantly in gene body regions.

## Discussion

While multiple studies have focused on the roles of canonical H3K4me3/H3K27me3 bivalent domains in the context of ES cell biology [3, 14, 29, 30], fewer studies have focused on identifying bivalent domains in committed lineages [7, 31]. Moreover, the presence of additional bivalent domains have not been thoroughly investigated in ES cells. In this study, we identified novel and distinct chromatin domains in ES cells consisting of the repressive histone modification, H4K20me3, paired with activating histone modifications, including H3K4me3, at promoter and intergenic regions, and H3K36me3, in gene body regions. We also found that the three histone modifications H3K4me3/H4K20me3/H3K36me3 co-occupy a subset of all H3K4me3 regions (5%), H4K20me3 regions (6%), and H3K36me3 (8%) regions. In addition, we observed the co-localization of the repressive histone modifications, H3K9me3 and H4K20me3, at these regions. There are several plausible explanations for co-localization of H4K20me3 and H3K9me3 in ES cells. First, H4K20me3 and H3K9me3 may function as redundant histone modifications that promote heterochromatin formation [17]. Second, dual marking of chromatin regions with H4K20me3 and H3K9me3 may facilitate interaction with a greater diversity of repressors relative to H4K20me3 or H3K9me3 alone. For example, H4K20me3 and H3K9me3 may regulate interactions between histone modifying enzymes and chromatin constituents. Along this line, the ESET, an H3K9 methyltransferase, interacts with several repressors including KAP1 and HP1, KAP1 associates with ESET and HP1 [32], and G9a interacts with HP1.

The intersection of H4K20me3 and H3K4me3 ChIP-Seq peaks was confirmed using reChIP-Seq, which allows for validation of co-enrichment of two histone modifications at the same loci [31, 33]. While our results strongly suggest that H4K20me3 co-localizes with H3K4me3 at specific loci, due to the resolution of sonicated chromatin utilized in the reChIP-Seq protocol (200-500 bp), it is possible that the two histone modifications are located on adjacent nucleosomes rather than on the same nucleosome.

We also found that H4K20me3 co-localizes with RNAPII/H3K4me3 at promoter and intergenic regions, and with elongating RNAPII (RNAPII-Ser2P)/H3K36me3 in gene body regions. Contrary to canonical H3K4me3/H3K27me3 bivalent domains, which are enriched at developmentally repressed genes in ES cells which are poised for activation upon differentiation [3], genes marked by H4K20me3/H3K4me3 or H4K20me3/H3K36me3 bivalent domains are largely active in ES cells. These findings suggest that H4K20me3 may positively regulate expression of a subset of target genes. In addition, we found that genes containing H4K20me3/H3K4me3 marks exhibit decreased RNAPII pausing relative to genes containing H3K4me3 marks, suggesting that dual marking of H4K20me3/H3K4me3 may regulate RNAPII pausing. Alternatively, H4K20me3 may limit expression of a subset of target genes to prevent their overexpression by dampening their transcriptional output. While a role for histone modifications in transcriptional dampening has been observed for H3K36me3 [34], the role for H4K20me3 in transcriptional dampening has not been fully described.

Bivalent H4K20me3/H3K4me3 or H4K20me3/H3K36me3 domains may also mark genes that are poised for repression upon differentiation. Along this line, while expression of H4K20me3/H3K4me3 co-marked genes is highly enriched in undifferentiated ES cells, few lineage committed cells express high levels of these genes (e.g. testis, thymocytes) (Fig. 3e). These results suggest that H4K20me3/H3K4me3 or H4K20me3/H3K36me3 domains support expression of ESC-specific genes and preserve their repression upon lineage-specific differentiation. In support of this model, our analyses suggest that dual marking by H4K20me3/H3K4me3 poises ESC-genes for rapid deactivation of RNAPII binding during differentiation.

A possible explanation for the co-occurrence is that H4K20me3 and H3K4me3 or H3K36me3 may serve as a unique set of markers to facilitate expression of a subset of genes in ES cells. Dual marking of repressive and activating histone modifications may allow for interaction with a broader set of transcriptional regulators.

Since H4K20me3 is known to be enriched in LTR and LINE repetitive sequences [2, 17], we evaluated the enrichment of repetitive DNA sequences in H4K20me3/H3K4me3 and H4K20me3/H3K36me3 bivalent domains. Our results also demonstrate that H4K20me3 marked bivalent domains are enriched at repetitive DNA elements in ES cells, where H4K20me3/H3K4me3 domains are enriched with LINE/L1 repeats. Moreover, we found that H4K20me3/H3K36me3 domains are enriched with LTR/ERK repeats. Bivalent marking of LTR and LINE repeats may prevent their activation during differentiation. Along this line, depletion of a H4K20 histone methyltransferase in ES cells resulted in decreased H4K20me3 and de-repression of LTR/LINE repeats in ES cells [17].

## Conclusions

Here, we describe novel bivalent domains containing the repressive H4K20me3 histone modification and activating H3K4me3 or H3K36me3 histone modifications at active genes in ES cells. Our results demonstrate that H4K20me3 pairs with the activating histone modification H3K4me3 and RNAPII at TSS regions, and with H3K36me3 in gene body regions of active genes. Moreover, while conventional H3K4me3/H3K27me3 bivalent domains mark developmental genes that are repressed in ES cells but poised for activation during differentiation, our model suggests that the novel H3K4me3/H4K20me3 bivalent domain marks genes that are poised for deactivation of RNAPII binding during differentiation. These results provide novel insight into the epigenetic landscape that supports self-renewal and differentiation of ES cells.

## Methods

### ES cell culture

R1 ES cells were cultured as previously described with minor modifications [4, 18, 19]. Briefly, R1 ES cells were obtained from ATCC in 2011 and cultured on irradiated MEFs in DMEM, 15% FBS media containing LIF (ESGRO) at 37 °C with 5% CO_2_. For ChIP experiments ES cells were cultured on gelatin-coated dishes in ES cell media containing 1.5 μM CHIR9901 (GSK3 inhibitor) for several passages to remove feeder cells. ES cells were passed by washing with PBS using serological pipets (sc-200,278, sc-200,280), and dissociating with trypsin.

### ChIP-Seq

ChIP-Seq was performed as previously described with minor modifications [4, 17, 18]. The rabbit polyclonal H3K36me3 (ab9050) antibody was obtained from Abcam. Briefly, 15 million mouse ES cells (R1) were harvested and chemically crosslinked with 1% formaldehyde (Sigma) for 8 min at 37 °C and subsequently sonicated. Sonicated cell extracts equivalent to 5 × 10^6^ cells were used for ChIP assays. ChIP-enriched DNA was end-repaired using the End-It DNA End-Repair kit (Epicentre), followed by addition of a single A nucleotide, and ligation of custom Illumina adapters. PCR was performed using Phusion High Fidelity PCR master mix. ChIP libraries were sequenced on Illumina HiSeq platforms according to the manufacture’s protocol. We also analyzed public H3K4me3 (GSE53093) and H4K20me3 (GSE94086) ChIP-Seq data, which we previously generated [17]. Sequence reads were mapped to the mouse genome (mm9) using bowtie2 [35]. ChIP-Seq read enriched regions were identified by SICER [36] with a window size setting of 200 bps, a gap setting of 400 bps and a FDR setting of 0.001. At least two ChIP-Seq biological replicates were performed. We have also applied the Kolmogorov–Smirnov test to obtain *p*-value statistics and compare densities at genomic regions and at SICER-peaks.

### reChIP-Seq

reChIP, also termed sequential ChIP, was performed as previously described with minor modifications [3, 17]. The H3K4me3 antibody (17–614) and the H4K20me3 antibody (07–463), were obtained from Millipore. Briefly, ES cells were harvested and chemically crosslinked with 1% formaldehyde (Sigma) for 5–10 min at 37 °C and subsequently sonicated. Sonicated cell extracts were used for ChIP assays. Cross-linked chromatin from ES cells was immunoprecipitated with antibodies against either H4K20me3 or H3K4me3 as described previously for ChIP-Seq [4, 17, 18], except that chromatin was eluted in a TE solution containing 20 mM DTT, 500 mM NaCL, and 1% SDS at 37° for 20 min. The eluted DNA was diluted 50-fold and a second round of immunoprecipitations was performed against the H3K4me3 or H4K20me3 antibody as described above.

reChIP-enriched DNA was end-repaired using the End-It DNA End-Repair kit (Epicentre), followed by addition of a single A nucleotide, and ligation of PE adapters (Illumina) or custom indexed adapters. PCR was performed using Phusion High Fidelity PCR master mix. reChIP libraries were sequenced on an Illumina HiSeq platform according to the manufacture’s protocol. Sequence reads were mapped to the mouse genome (mm9) using bowtie2 [35]. reChIP-Seq read enriched regions were identified by SICER [36] with a window size setting of 200 bps, a gap setting of 400 bps and a FDR setting of 0.001. For a comparison of ChIP-enrichment between samples a fold-change threshold of 1.5 and an FDR setting of 0.001 were used. The RPBM measure (read per base per million reads) or RPKM measure (read per kilobase per million reads) was used to quantify the density of histone modification occupancy at genomic regions from ChIP-Seq datasets. Bedtools [37] intersect was used to evaluate overlaps between histone modification occupancy.

### RNA-Seq analysis

The RPKM measure (read per kilo bases of exon model per million reads) proposed previously [38] was used to quantify the mRNA expression level of a gene from RNA-Seq data sets. Differentially expressed genes were identified using EdgeR (FDR < 0.001 & FC > 2) [39]. Genes with RPKM < 3 in both conditions in comparison were excluded from this analysis.

## Abbreviations

ChIP: Chromatin immunoprecipitation
ChIP-Seq: Chromatin immunoprecipitation followed by sequencing
CTD: c-terminal domain
EB: Embryoid body
ES cell: Embryonic stem cell
GSEA: Gene set enrichment analysis
H3K27me3: Histone 3, lysine 27 trimethylation
H3K36me3: Histone 3, lysine 36 trimethylation
H3K4me3: Histone 3, lysine 4 trimethylation
H3K9me3: Histone 3, lysine 9 trimethylation
H4K20me3: Histone 4, lysine 20 trimethylation
MEF: Mouse embryonic fibroblast
NELF: Negative elongation factor
NP: Neural progenitor
reChIP: Sequential ChIP
RNAPII: RNA polymerase II
RNA-Seq: RNA sequencing
RPKM: Read per kilo bases of exon model per million reads
TI: Traveling index
TSS: Transcription start site
